# Werner syndrome: a model for sarcopenia due to accelerated aging

**DOI:** 10.18632/aging.101265

**Published:** 2017-07-19

**Authors:** Masaya Yamaga, Minoru Takemoto, Mayumi Shoji, Kenichi Sakamoto, Masashi Yamamoto, Takahiro Ishikawa, Masaya Koshizaka, Yoshiro Maezawa, Kazuki Kobayashi, Koutaro Yokote

**Affiliations:** ^1^ Department of Clinical Cell Biology and Medicine, Chiba University Graduate School of Medicine, Chiba 260-8670, Japan; ^2^ Department of Medicine, Division of Diabetes, Metabolism and Endocrinology, Chiba University Hospital, Chiba 260-8670, Japan; ^3^ School of Medicine, International University of Health and Welfare, Department of Diabetes, Metabolism and Endocrinology, Chiba 286-8686, Japan; ^4^ Eastern Chiba Medical Center, Chiba 283-8686, Japan; ^5^ Asahi General Hospital, 1326 I, Chiba 289-2511, Japan

**Keywords:** sarcopenia, progeria, Werner syndrome

## Abstract

Werner syndrome (WS) is a rare inheritable progeroid syndrome caused by a mutation in the WRN gene. Although WS has been described as a characteristic appearance of very slender extremities with a stocky trunk, few studies have investigated the loss of muscle mass, fat mass distribution (body composition), and mobility according to age and sex. Therefore, the aim of this study was to precisely describe the body composition in WS. Nine Japanese patients with WS (four males and five females; mean age 48±8.8 years) were recruited. Body composition was examined by dual-energy X-ray absorptiometry and computed tomography (CT). The hand grip strength and mobility were evaluated using the two-step test, stand-up test and 25-question geriatric locomotive function scale. The mean skeletal muscle index (SMI) was 4.0±0.6 kg/m. SMI of all patients met the criteria of sarcopenia, even though some patients were aged < 40 years. All patients also showed deceased mobility. In conclusion, these results indicate that all patients with WS, even those aged < 40 years, had already lost muscle mass to the level of sarcopenia. Continued research on sarcopenia in WS might facilitate the discovery of novel mechanisms and development of new treatment strategies for sarcopenia.

## INTRODUCTION

Werner syndrome (WS) is a representative hereditary progeroid syndrome [1] with a high incidence in Japan [2]. It is frequently accompanied with metabolic disorders, such as diabetes and dyslipidemia [3, 4], vascular disease [5], and malignancy [6]. Patients with WS also show characteristic appearances of short stature, light body weight, and a so called bird-like face in which the nasal bridge appears to be pinched and the subcutaneous tissue is diminished [7]. Physicians with experience in treatment of WS may doubt the presence of the disease even if the patient presents with a characteristic bird-like face and a high pitched, squeaky, and/or hoarse voice. The skin of these patients is usually atrophic and tight. Clavus, callus, or intractable ulcers of the feet are also frequently observed [8]. Patients with WS have very slender extremities and have been described as a dried tree with a stocky trunk [7]. The appearance of these patients is sometimes referred to as Cushing or Klinefelter syndrome-like central obesity, although a few reports describe anomalies of muscle mass, fat mass distribution (body composition), and mobility, which differ according to age and sex. Therefore, the aim of this study was to precisely describe the body compositions of patients with WS.

## RESULTS

### Sarcopenia is a characteristic clinical feature of WS that appears before visceral fat accumulation

**Figure 1:**
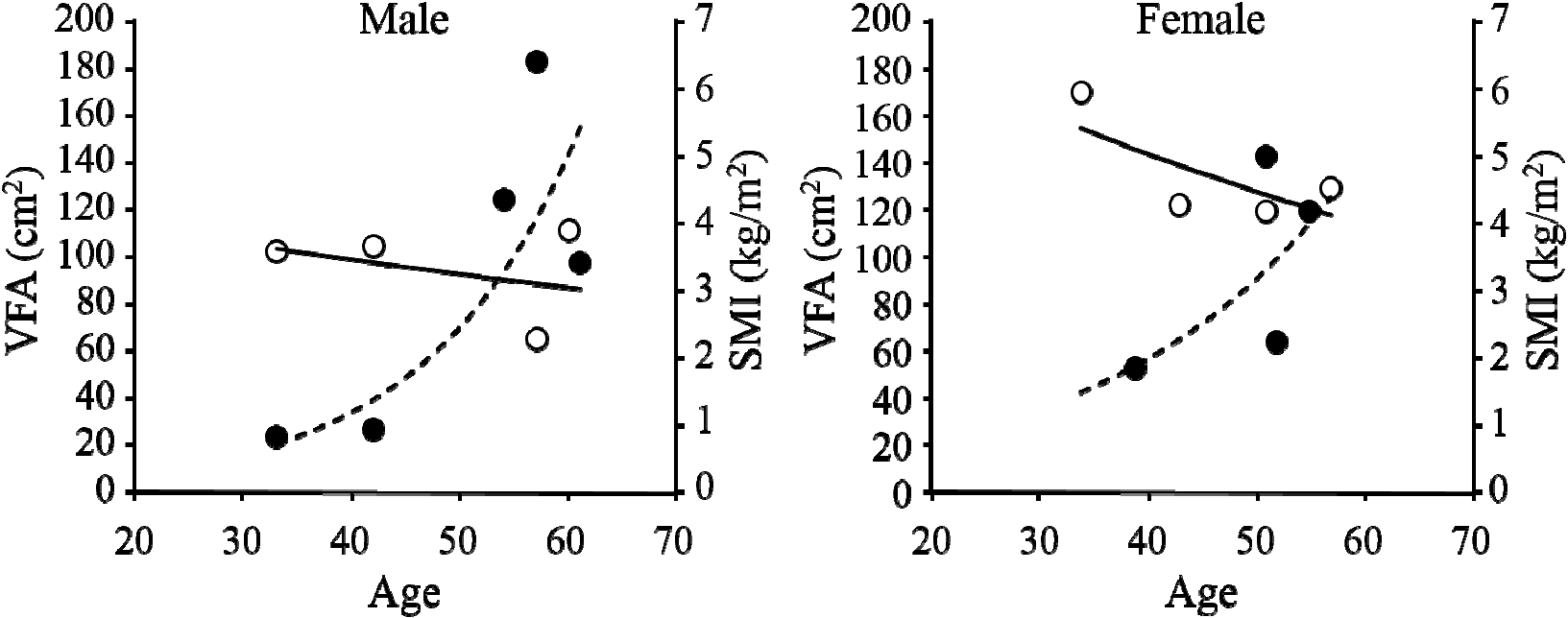
Correlations among skeletal muscle index (SMI), visceral fat area (VFA), and age Values for SMI and VFA were plotted against age. SMI is indicated by open circles and VFA is indicated by closed circles. Each line and the dotted line indicate an approximate curve.

The basic characteristic of the patients is shown in Table 1. The body weight and body mass index (BMI) of all patients were low. According to the diagnostic criteria of the European Working Group on Sarcopenia in Older People [9], sarcopenia is diagnosed as hand grip strength and skeletal muscle index (SMI), as evaluated by dual-energy X-ray absorptiometry (DXA), of less than 26 kg and 7.0 kg/m^2^ for males and 18 kg and 5.4 kg/m^2^ for females, respectively. All patients were diagnosed with sarcopenia even though some were aged <40 years. SMI measurements were plotted against age (Figure 1). Although SMI usually decreases with age, patients with WS rapidly loose muscle mass and most of the participants in this study had already lost muscle mass to the level of sarcopenia. A graph of the accumulation of showed that sarcopenia seemed to appear before VFA accumulation.

**Table 1:** Basic patient characteristics

Patient number	1	2	3	4	5	6	7	8	9	Mean±SD
Age (year)	56	60	40	60	39	51	42	43	41	48±8.8
Sex	M	F	M	F	F	M	F	M	F	
mutation	6/6	6/6	4/11	4/4	11/11	4/7	4/4	4/4	4/4	
Height (cm)	162.8	148	167.1	146.4	148.6	164	146.8	161.3	145.5	154.5±8.9
BW (kg)	46	40.8	48	43	33.6	51.8	31.4	43	36	41.5±6.8
BMI (kg/m^2^)	17.4	18.6	17.2	20.1	15.2	19.3	14.6	16.5	17.0	17.3±1.8
SMI (kg/m^2^)	4.5	3.9	5.1	3.4	3.3	4.2	3.7	4.3	N/A	4.0±0.6
HGS (kg)	26.3	11.4	29	6.9	13.2	19.3	14.4	17.8	12.6	16.6±7.2
VFA (cm^2^)	118.9	102.8	52.5	183.0	25.6	142.0	27.5	N/A	N/A	93.2±60.2
BMD (L) (YAM)	70	78	107	79	97	93	88	85	N/A	87.1±11.8
BMD (F) (YAM)	70	57		68	74	60	76	81	N/A	69.4±8.6
DM	-	+	-	+	-	+	-	-	+	
HT	-	+	+	-	-	+	-	-	-	
DL	+	+	+	-	-	+	-	-	-	

BW: body weight, BMI: body mass index, SMI: skeletal muscle index, HGS: hand grip strength, VFA: visceral fat area, BMD (L): bone mineral density (lumbar spine), BMD (F): bone mineral density, YAM: young adult mean, DM: diabetes mellitus, HT: hypertension, DL: dyslipidemia, SD: standard deviation

### WS patients exhibited lower mobility

According to the Japanese Orthopedic Association, any of the following criteria meet a diagnosis of decreased mobility [10]: two-step score < 1.1; difficulty in standing from a 20-cm-high seat using both legs in the stand-up test, or 25-question GLFS score ≥ 16. As shown in Table 2, scores of all patients with WS meet the criteria of impaired mobility. Correlation between body composi-tion and metabolic parameters was also investigated.

**Table 2:** The assessment of muscle strength and mobility in patients with WS

Patient number	1	2	3	4	5	6	7	8	9
Two-step test	0.52	0.79	0.81	0.41	1.27	N/A	1.03	N/A	1.18
Stand-up test	Bilateral 40cm	Bilateral 30cm	Bilateral 40cm	N/A	One leg 40cm	N/A	Bilateral 10cm	N/A	One leg 40cm
25-GLFS score	73	22	29	63	N/A	54	N/A	57	53

For this purpose, patients were divided into two groups: those with and without diabetes. As shown in Table 3, patients with diabetes had a greater BMI and accumulation of VFA, while there was no difference in SMI between groups.

**Table 3:** Differences in clinical parameters among patients with WS with or without diabetes

	Without diabetes	n	Diabetes	n	p value
Age (year)	44±6.9	5	53±9.1	4	0.16
25-question GLFS score	40±31.7	4	43±18.8	4	0.88
Two-step test value	0.73±0.49	5	0.60±0.51	4	0.71
Grip strength (kg)	20.1±7.1	5	12.5±5.1	4	0.11
VFA (cm^2^)	56.1±43.6	4	142.6±40.1	3	0.04*
SMI (kg/m^2^)	4.2±0.7	5	3.8±0.4	3	0.4
BMD (L) (YAM)	89.4±13.8	5	83.3±8.4	3	0.47
BMD (F) (YAM)	75.3±4.6	4	61.7±5.7	3	0.03*
BW (kg)	40.4±7.5	5	42.9±6.6	4	0.61
BMI (kg/m^2^)	16.2±1.2	5	18.7±1.3	4	0.02*
Adiponectin (ng/mL)	6.4±2.8	4	6.6±4.1	4	0.95
TNF-α (pg/mL)	1.4±0.6	4	3.0±4.3	4	0.51
Leptin (ng/nL)	7.2±3.6	4	30.0±16.9	4	0.07

GLFS: geriatric locomotive function scale, VFA: visceral fat area, SMI: skeletal muscle index, BMD (L): bone mineral density (lumbar spine), BMD (F): bone mineral density (femoral neck), YAM: young adult mean, BW: body weight BMI: body mass index, TNF: tumor necrosis factor, * p <0.05

## DISCUSSION

This is the first report to precisely evaluate the body composition and mobility of patients with WS of different ages and both sexes. All patients met the criteria for sarcopenia even though some were aged <40 years and sarcopenia seemed to appear before the accumulation of VFA.

The dramatic increase in the number of elderly people is a significant public health concern, particularly in developed countries. As the number of elderly people continues to increase, sarcopenia, a progressive loss of muscle and strength, is certain to become a huge medical problem because the disease is associated with the deterioration of skeletal muscle function, which leads to disability, frailty, and increased morbidity and mortality [9, 11].

Although the underlying mechanism remains largely unknown, recent studies have revealed that sarcopenia is characterized by low physical activity [12]; poor nutrition, particularly with a low protein diet [13]; chronic low-grade systematic inflammation, known as “inflammaging” [14]; lipotoxicity [15]; dysfunction of neuromuscular junctions [16]; decreased blood concentrations of sex hormones [17] and 25(OH) vitamin D3 [18]; and stem cell aging [19].

WS, also known as adult progeria, is an autosomal recessive disorder caused by a mutation in the gene encoding RecQ DNA helicase [20]. Because some aging phenotypes accelerate WS, this disease has been used as a model of human aging. The results of this study confirmed that patients with WS aged <40 years have already lost skeletal muscle mass and fulfilled the diagnosis of sarcopenia. Loss of skeletal muscle mass usually begins in the fifth decade of life in the general population, while aging of the skeletal muscle is indeed accelerated in WS.

The underlying mechanism of sarcopenia in WS also remains incompletely understood. It has been reported that mammalian target of rapamycin (mTOR) signaling by fibroblasts is dysregulated in WS [21]. Because mTOR signaling is important for muscle hypertrophy, dysregulated mTOR signaling might play a role in sarcopenia development [22]. It has also been reported that mesenchymal stem cells, which are derived from pluripotent (iPS) cells, have a senescence phenotype in WS [23]. Therefore, it is also possible that satellite cells within the skeletal muscle, which are a type of stem cell, have an aging phenotype and low capacity for regeneration of the skeletal muscle. Using peripheral blood cells sample, it has recently been reported that WS is associated with intrinsic and extrinsic epigenetic age acceleration [24]. Therefore, accelerated epigenetic aging may result in accelerated sarcopenia seen in WS.

It is also intriguing that the sarcopenia seemed to appear before the accumulation of VFA. It has been reported that the accumulation of VFA seen in WS was related with metabolic disorders such as diabetes and dyslipidemia due to high insulin resistance [3, 25]. This is also true in our study, because WS with diabetes exhibited more VFA than WS without diabetes. Although we do not know the precise mechanisms by which accumulation of VFA was accelerated in WS, reduced physical activity due to the sarcopenia may affect accumulation of VFA.

Several interventions for sarcopenia have been reported, including diets rich in branched amino acids [26], exercise [27], and supplementation of vitamin D [28] as well as growth [29] and sex hormones [17]. Because WS is reportedly linked to a predisposition for malignancy, supplementation of growth and sex hormones might not be a good option for sarcopenia in WS. WS is highly associated with osteoporosis. It has recently been reported that femoral osteoporosis is more common in patients with WS [30]. Our results also indicated that the WS patients with diabetes seemed accelerated femoral osteoporosis than lumbar osteoporosis (Table 3). Although the mechanisms how osteoporosis is accelerated in WS have not been fully understood yet, it has been reported that osteoblastic bone formation was impaired in the patients [31]. Further analyses of the mechanism of osteoporosis seen in patients with WS may enable us to understand the mechanism of osteoporosis seen in normal aging. Nevertheless, patients with WS exhibit osteo-sarcopenic obesity; therefore, vitamin D supplementation may be a good option for treating osteoporosis and sarcopenia.

There were several limitations in this study that should be addressed. Although this study included patients of different ages and both sexes, this was cross-sectional study with a small number of patients. To overcome this problem, a registry of Japanese patients with WS was recently started by the Japan Agency for Medical Research and Development [http://www.m.chiba-u.jp/class/clin-cellbiol/werner/index.html]. A case of WS was also recently reported in a 17-year-old [32]. Hence, long-term follow-up in young patients with WS will allow further investigations of the precise natural history of WS.

In conclusion, WS is a human model of sarcopenia and continued research of sarcopenia in WS might facilitate the discovery of novel mechanisms and development of new treatment strategies for sarcopenia in WS as well as natural aging.

## MATERIALS AND METHODS

### Subjects

The study cohort included nine outpatients with WS who underwent treatment at the Chiba University Hospital (Chiba, Japan). The study protocol was approved by the ethics committee of Chiba University Hospital and was conducted in accordance with the ethical principles of the Declaration of Helsinki. All patients were informed of the study aims and methods and provided written, informed consent before participation in the study.

### Genotyping

Genomic DNA was extracted from anonymized blood samples collected in tubes containing ethylene-diaminetetraacetic acid/disodium salt using the QIAamp DNA Blood Mini Kit. For genetic testing, exons were individually amplified as described elsewhere [20, 33] and then sequenced.

### Measurement of body composition

Body composition was examined by dual-energy X-ray absorptiometry (DXA) [34]. The visceral fat area (VFA), as reference of abdominal obesity, was evaluated by computed tomography [35].

### Assessment of muscle strength and mobility

Hand grip strength: Hand grip strength of the non-dominant hand was measured using a digital hand dynamometer (TKK5401; Takei Scientific Instruments, Niigata, Japan). The mean value of three measurements was used for analysis.

Two-step test: The two-step test was performed as previously described [36] as a measure of stride length to assess walking ability, muscle strength, balance, and flexibility. The two-step test score was calculated using the following formula: the length of two steps (cm)/height (cm).

Stand-up test: The stand-up test was also performed as previously described [37] as a measure of leg strength when standing up with one or both legs from a specified height of 10, 20, 30, and 40 cm. If the patient could stand without leaning back and maintain a standing posture for 3 s, then the patient was considered as having achieved that height level.

The 25-question geriatric locomotive function scale (GLFS): The 25-question GLFS was employed as described previously 38 and scored with a five-point scale, where a higher score indicated poorer locomotion.

### Statistical analysis

Comparisons between two groups were conducted using Welch's *t*-test. A probability (*p*) value of <0.05 was considered statistically significant. All statistical analyses were performed using JMP pro12 software (SAS Institute Japan, Tokyo, Japan).

## Abbreviations

WS: Werner syndrome
CT: computed tomography
SMI: skeletal muscle index
DXA: dual-energy X-ray absorptiometry
mTOR: mammalian target of rapamycin
VFA: visceral fat area
GLFS: geriatric locomotive function scale.
